# Clinical outcomes for T_1-2_N_0-1 _oral tongue cancer patients underwent surgery with and without postoperative radiotherapy

**DOI:** 10.1186/1748-717X-5-43

**Published:** 2010-05-27

**Authors:** Su Jung Shim, Jihye Cha, Woong Sub Koom, Gwi Eon Kim, Chang Geol Lee, Eun Chang Choi, Ki Chang Keum

**Affiliations:** 1 Department of Radiation Oncology, Eulji Hospital, Eulji Medical Center, Seoul, Korea; 2 Department of Radiation Oncology, Yonsei Cancer Center, Yonsei University Health System, Seoul, Korea; 3 Department of Otorhinolaryngology, Yonsei Cancer Center, Yonsei University Health System, Seoul, Korea

## Abstract

**Background:**

The aim of this study was to assess the results of curative surgery with and without radiotherapy in patients with T_1-2_N_0-1 _oral tongue squamous cell carcinoma (OSCC) and to evaluate survival and prognostic factors.

**Methods:**

Retrospective analysis of 86 patients with T_1-2_N_0-1 _OSCC who received surgery between January 2000 and December 2006. Fourteen patients (16.3%) received postoperative radiotherapy (PORT). Patient characteristics, tumor characteristics, treatment modality, failure patterns, and survival rates were analyzed.

**Results:**

The median follow-up was 45 months. The five-year overall survival (OS) and disease-free survival (DFS) rates were 80.8% and 80.2%, respectively. Higher tumor grade and invasion depth ≥ 0.5 cm were the significant prognostic factors affecting five-year OS and DFS (OS rate; 65% vs. 91%, p = 0.001 for grade; 66% vs. 92%, p = 0.01 for invasion depth: DFS rate; 69% vs. 88%, p = 0.005 for grade; 66% vs. 92%, p = 0.013 for invasion depth). In the risk group, there was no local failure in patients with postoperative radiotherapy.

**Conclusions:**

In T_1-2_N_0-1 _OSCC, factors that affected prognosis after primary surgery were higher tumor grade and deep invasion depth over 0.5 cm. Postoperative radiotherapy should be considered in early oral tongue cancer patients with these high-risk pathologic features.

## Background

The oral tongue is the most common subsite for squamous cell carcinoma of the oral cavity, excluding the lip [1]. In advanced cases, surgical resection followed by radiotherapy (RT) with or without chemotherapy is performed, and it seems to be beneficial. In early cases (T1-2), surgery is often the preferred form of treatment [2]. However, the management of cervical lymph nodes (LN) and the role of postoperative adjuvant treatment remain uncertain.

Although surgery has emerged as the preferred initial treatment approach for the majority of patients with tumors of the oral cavity, adjuvant postoperative radiation is commonly recommended to enhance the likelihood of locoregional tumor control [3]. The results of two randomized trials suggest that postoperative chemoradiation may be beneficial in improving local-regional control and disease-free survival among patients selected for specific high-risk features of head and neck cancer [4,5]. As with other head and neck cancers, postoperative radiation therapy (PORT) in oral tongue squamous cell carcinoma (OSCC) is recommended for patients with large primary tumors (T3, T4), close or positive surgical margins, and evidence of perineural invasion (PNI), multiple positive nodes, or extracapsular extension (ECS). Data were limited for high-risk features of recurrence and PORT in early-stage OSCC. Furthermore, most series reported a mixed patient population with oral cavity cancer [6,7].

Because of the extremely low salvage rate of recurred oral tongue cancer [8], the proper extent and modality of initial treatment is very important. This study retrospectively reviewed patients with oral tongue cancer treated at the Yonsei University Health System in Seoul, Korea, to investigate the pathologic prognostic factors in patients with T_1-2_N_0-1 _OSCC in terms of disease-free survival (DFS) and overall survival (OS), and to verify the role of PORT in patients with a high risk of recurrence.

## Methods

Between 2000 and 2006, 234 patients with oral tongue cancer were treated at the Yonsei University Health System, Seoul, Korea. Among them, 90 (38.5%) were treated surgically at stage T1-2N0-1. Of these, one patient presented with myoepithelial carcinoma, and one patient with adenoid cystic carcinoma. One patient who received neoadjuvant chemotherapy before surgery and one patient who received chemotherapy for acute lymphoblastic leukemia before the diagnosis of oral tongue cancer were eliminated from the analysis. Finally, the medical records of 86 patients were retrospectively reviewed with institutional review board (IRB, Severance Hospital, Yonsei University Health System) approval. Tumor staging was based on the pathology findings, according to the American Joint Committee on Cancer Staging System, 6^th ^edition. In addition, the following variables were recorded: size and invasion depth of the primary tumor (tumor thickness), grade of differentiation, status of resection margins, lymphovascular invasion, and perineural invasion. The grade of differentiation was also divided into two groups: well-differentiated and moderate-to-poorly differentiated. To determine the status of resection margins, the closest were labeled as positive for a margin invaded by cancer cells, negative for a safety margin not less than 0.5 cm, and close for the safety margin less than 0.5 cm. The pathologically proven metastatic lymph node was evaluated by level, diameter, and perinodal extension.

All patients received surgery for the primary site and neck. Resection of the primary site was grouped by the extent of the resection as simple excision, hemiglossectomy, and wide excision. None of the patients underwent a total glossectomy. Neck node dissection was performed in 64 patients. The Type of neck dissection used was elective supraomohyoid except 4 cases of modified radical neck dissection. Fourteen patients received PORT. Because this was a retrospective study, the indication for RT had been determined by the individual physician. Follow-up time was calculated from the date of the cancer operation until the date of the last hospital visit, admission, or death, and each event-free survival was calculated from the date of the cancer operation to the date of diagnosis of each event. The five-year disease-free survival (DFS) rate, local recurrence-free survival (LRFS) rate, regional recurrence-free survival (RRFS) rate, distant metastasis--free survival (DMFS) rate, and overall survival (OS) rate were calculated using the Kaplan-Meier method. The differences in survival rates were compared by the log-rank test. Prognostic factors influencing survival were analyzed by univariate and multivariate analyses using Cox's proportional hazards model. A *p *value ≤ 0.05 was considered statistically significant.

## Results

### Patient and tumor characteristics

Patient characteristics are listed in Table 1. Ages ranged from 23 to 82 years, with a median of 54 years. All of the patients showed an Eastern Cooperative Oncology Group (ECOG) performance status 0 or 1. There were 50 (58%) patients with stage I, 22 (26%) with stage II, and 14 (16%) with stage III disease. Pathologic specimens were graded as well, moderately, or poorly differentiated, according to the World Health Organization criteria. Fifty-two patients (61%) had well-differentiated disease, and 34 (39%) moderate-to-poorly differentiated disease. Among the total of 86 patients, 14 patients (16%) received PORT. Of the 14 patients, 10 received neck node RT by bilateral neck node irradiation. The average dose to the primary area was 59.7Gy, involved neck area was 57.1Gy, and elective neck area was 45.6Gy. In case of close or positive margin, 60-65 Gy was given. Seven patients of 14 patients (50%) received 3-dimensional conformal therapy or intensity modulated radiation therapy.

**Table 1:** Patient's characteristics (n = 86)

Characteristics		No. of cases	%
Age (year)	< 55	38	44
	≥ 55	48	56
Gender	Male	48	56
	Female	38	44
Performance	ECOG 0	23	27
	ECOG 1	63	73
T classification	T1	55	64
	T2	31	36
N classification	N0	72	84
	N1	14	16
Stage	I	50	58
	II	22	26
	III	14	16
Grade	Well	52	61
	Moderate-poor	34	39
Invasion depth (cm)	≤ 0.5	48	56
	> 0.5	38	44
Resection margin	Negative	65	76
	Close (< 0.5 cm)	19	22
	Positive	2	2
Lymphovascular invasion	Yes	3	4
	No	83	96
Perineural invasion	Yes	3	4
	No	83	96
Radiotherapy	Yes	14	16
	No	72	84
Op of primary site	Simple excision	11	13
	Hemiglossectomy	66	77
	Wide excision	9	11
Neck dissection	Yes	64	74
	No	22	26

*Abbreviations *: ECOG = Eastern Cooperative Oncology Group.

### Survival and prognostic analysis

**Figure 1:**
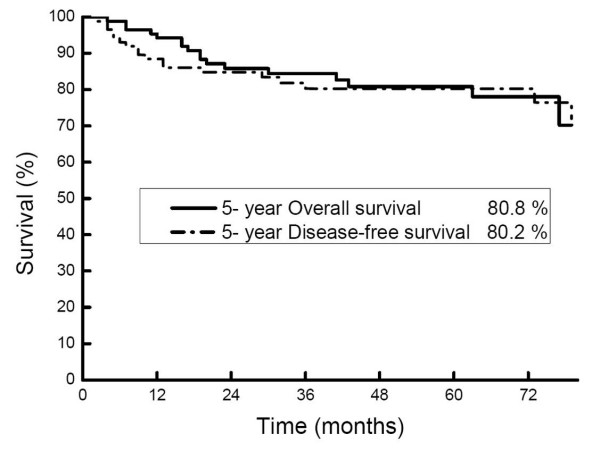
**Kaplan-Meier plots of overall and disease-free survival**. Five-year survival rates were 80.8% and 80.2%, respectively

The median follow-up was 45 months (range: 4- 99 months). The five-year OS and DFS rates were 80.8% and 80.2%, respectively (Figure 1). By univariate analysis, grade of differentiation was determined to be the statistically significant prognostic factor for five-year DFS, LRFS, RRFS, DMFS, and OS rates. Invasion depth was a significant factor predictive of five-year DFS, RRFS, and OS rates (Table 2). By multivariate analysis regarding the OS and DFS rates, two factors proven to be significant by the univariate analysis were confirmed to be statistically significant (Table 3).

**Table 2:** Univariate analysis of five-year disease-free survival (DFS), local recurrence-free survival (LRFS), regional recurrence-free survival (RRFS), distant metastasis-free survival (DMFS) rate, and overall survival (OS) rate

	5-year DFS		5-year LRFS		5-year RRFS		5-year DMFS		5-year OS	
	
	(%)	*p *value	(%)	*p *value	(%)	*p *value	(%)	*p *value	(%)	*p *value
Age (year)		0.233		0.239		0.712		0.312		0.789
< 55	84		97		84		89		84	
≥ 55	77		87		78		93		77	
Performance		0.718		0.162		0.44		0.692		0.421
ECOG 0	87		100		87		87		87	
ECOG 1	77		88		78		94		78	
T classification		0.331		0.575		0.101		0.856		0.186
T1	84		91		85		96		86	
T2	74		94		73		83		71	
N classification		0.725		0.963		0.52		0.856		0.186
N0	81		92		82		91		83	
N1	79		93		79		92		67	
Stage		0.568		0.685		0.218		0.228		0.151
I	85		90		86		96		87	
II	72		96		72		82		73	
III	79		93		79		92		67	
Grade		0.005		0.015		0.013		0.024		0.001
Well	88		95		89		96		91	
Moderate-poor	69		86		69		84		65	
Invasion depth (cm)		0.018		0.166		0.008		0.201		0.023
≤ 0.5	92		98		92		96		92	
> 0.5	66		83		66		87		66	
Resection margin		0.186		0.624		0.081		0.292		0.360
Negative	84		92		86		94		84	
Close (< 0.5 cm) or positive	65		89		62		86		67	
Lymphovascular invasion		0.358		0.686		0.292		0.043		0.586
Yes	67		100		67		67		50	
No	81		91		82		93		82	
Perineural invasion		0.662		0.135		0.598		0.602		0.283
Yes	67		67		50		100		67	
No	81		93		82		91		81	
Radiotherapy		0.475		0.161		0.775		0.548		0.647
Yes	86		100		86		86		86	
No	79		90		80		93		80	
Operation of primary site		0.284		0.608		0.775		0.548		0.647
Simple excision	74		83		74		100		81	
Hemiglossectomy	78		82		80		89		81	
Wide excision	100		100		100		100		100	
Neck dissection		0.534		0.556		0.518		0.898		0.882
Yes	82		93		83		92		81	
No	76		88		75		90		80	

*Abbreviations *: ECOG = Eastern Cooperative Oncology Group

**Table 3:** Multivariate analysis of overall survival and disease-free survival rate

	Overall survival rate			Disease-free survival rate		
		
	HR	(95% CI)	*p *value	HR	(95% CI)	*p *value
Grade (well vs. mod-poor)	6.93	(2.23-21.56)	0.001	4.16	(1.55-11.18)	0.005
Invasion depth (≤ 0.5 vs. > 0.5)	3.94	(1.39-11.14)	0.01	3.51	(1.31-9.46)	0.013

*Abbreviations*: HR = hazard ratio; CI = confidence interval.

### Patterns of failure

The 18 cases of recurrence in total included 8 local failures, 15 regional failures, and 8 distant metastases. Five patients showed local and regional failures, 7 showed regional and distant failures, and one patient showed local and distant failures. Of those patient with initial recurrences, salvage treatment was attempt in 15 patients. The operation was performed in 8 patients and 4 patients received PORT only and two patients received concurrent chemo-radiotherapy (CCRT). Three patients received CCRT without the operation, two received chemotherapy only and two received RT only. Of the 18 patients of recurrence cases, 13 died. Of patients who could undergo an operation by salvage treatment, 4 patients were successfully treated by salvage treatment and followed up as having no evidence of disease but 13 died of the disease and 1 survived with the disease during the follow-up period. Patients who showed only local failures did not die, and only those who showed regional failures or distant metastases died. The 14 patients who received RT did not show any local failure. However, two of them showed regional failures and died within six months after treatments that revealed regional failures and lung metastases. There were four intercurrent deaths. One patient died of end stage renal disease, one died of stomach cancer and remaining patients died of other chronic diseases.

### Risk group analysis

Fifty-seven patients had an invasion depth over 0.5 cm or a grade of moderate-to-poor, and all the recurrences were found in this group. These patients were divided into those who had received RT and those who had not. The recurrence rates of the two groups were reviewed, and the results are shown in Table 4. Although there was no statistically significant difference among the recurrence rates, there was no local failure in the group that received RT.

**Table 4:** Disease recurrence in the risk group according to radiation therapy

	Radiation therapy (n = 13)		No radiation therapy (n = 44)		*p *value
		
	No. of patients	%	No. of patients	%	
Local recurrence	0	0	8	18	0.107
Regional recurrence	2	15	13	29	0.252
Distant recurrence	2	15	6	14	0.591

Total recurrence	2	15	16	36	0.137

## Discussion

This study retrospectively observed the treatment results of patients with oral tongue cancer in relatively early stages corresponding to T_1-2_N_0-1_. Operative treatments have been primarily performed for early oral tongue cancers, and, although there have been some reports that five-year survival rates of stage I-II diseases were 80% or higher [9]. Rusthoven *et al*. reported the five-year survival and cause-specific survival rates of stage I and II oral tongue cancers as 60.9% and 83.5%, respectively, and in other oral cavity subsites as 64.7% and 94.1%, respectively, based on the patient SEER database [10]. Although the prognosis of oral tongue cancers was poorer than that of cancers in other oral cavity sites, in this study, the five-year OS and DFS were shown as 80.8% and 80.2%, respectively, far better than those from other reports. This is thought to be due to the appropriate RT performed in this institute against early tongue cancers along with operative treatments.

Although primary RT and surgery are potential treatment options for oral tongue cancer, most oral tongue cancers are treated surgically [1]. Currently, RT is often used in addition to surgery and tends to be given postoperatively, often because of unfavorable histology. Many oncologists would recommend adjuvant RT for large tumors if surgical margins are close to or involved with the tumor or after neck dissection where there are many positive nodes. In this study, RT was performed according to the opinions of surgeons, rather than to certain criteria, and, consequently, determining the role of RT was difficult. The 14 patients who were treated by RT constituted too small a sample for significant analysis. Thus, comparison of the results of patients who received RT with the results of those who did not may not be meaningful. However, no local failure occurred among patients who had exhibited risk factors and had received RT. Although this result was not indicated to be statistically significant because the number of patients was not large enough, it may become a finding helpful in performing radiation therapy against early tongue cancers later.

Recently, awareness of the frequency of occult node metastases in early tongue cancer has increased, and researchers have attempted to identify molecular markers predictive of occult node metastases [11]. The lymphatic system of the oral tongue shows extensive communication across the midline, so carcinomas of the oral tongue can metastasize bilaterally. The regional recurrence rate of the untreated N0 neck was found to be between 30 and 47% for early T1-T2 carcinoma [12], and which has led many authors to propose elective neck dissection. Many institutes have reported that improved neck control and increased survival rates have been achieved by adopting elective neck dissections [13]. Nodal recurrence in the contralateral neck is a significant cause of regional failure after elective ipsilateral neck dissection, and postoperative irradiation is recommended for cases of more than three positive nodes or in the presence of extra capsular invasion. Elective neck irradiation is advantageous in that it can be used as an alternative to neck dissections or to treat both sides of the neck after a neck dissection. In many reports, elective whole neck irradiation provided higher control rates, as compared to patients managed with limited or no neck treatment [14]. In the current study, among the 57 patients with risks, regional failures were observed in 13 patients of 44 (29%) with no neck irradiation and in 2 patients of 13 (15%) treated with neck irradiation. Although this result is not statistically significant, fewer regional failures occurred in cases where adjuvant RT was performed in the neck area. Studies to elucidate the role of RT in relation to regional recurrences, as well as local recurrences, should be continued.

Risk factors for recurrence in oral tongue cancer include an infiltrating pattern of tumor growth, diffuse histological invasion, and a tumor within 5 mm of the resection margin [15]. This study has retrospectively analyzed prognostic factors for patients with T_1-2_N_0-1 _OSCC treated primarily by surgery in an attempt to define more exactly a subgroup at high risk for recurrence. A number of histo-pathological parameters were evaluated to identify patients at high-risk for locoregional control and survival, including tumor grade, depth of invasion, tumor size, and the status of the resection margin. In this study, depth of invasion and tumor grade seemed to affect the DFS and OS rates. Al-Rajhi *et al*. reported that tumor thicknesses affected prognoses, and that lesions less than 10 mm had remarkably favorable prognoses [16]. However, the critical tumor thickness limit varied from 2 to 10 mm in different studies [15]. There is no agreement on the appropriate tumor thickness below which elective treatment should not be recommended. With regard to tumor grade, Arduino *et al*. reported that histological grading was related, as an independent factor, in predicting survival in patients with oral squamous cell carcinoma [17]. In this study, tumor grades were shown to be factors related to OS and DSF rates. Therefore, aggressive treatments should be considered for patients with these risk factors.

Although the combination of chemotherapy with surgery and RT has improved cure rates in some other head and neck cancers, its role in the management of oral cavity tumors is not clear. Some advocate its use in young patients, when there are multiple involved cervical nodes, and in the presence of adverse histological features [5]. Although no survival benefit has been confirmed to date, the results of studies involving large series are awaited. The past decade has seen the advent of intensity-modulated radiotherapy (IMRT) to treat head and neck cancer. Toxicity and locoregional control rates have been promising [18,19]. Gomez *et al*. advocate using IMRT when available for all patients treated in the postoperative setting for oral cavity, because acceptable acute toxicity of normal structures has been found with at least similar (if not superior) outcomes for local control [20]. In this study, of 14 patients who received RT, three received IMRT. Although it was difficult to analyze toxicity due to the limitation of the retrospective study, the patients who received IMRT showed tolerable toxicity. Radiation to the oral cavity can develop complications and affect patients' quality of life. Further studies with larger numbers of patients are necessary and should include the follow-up data of complications in addition to the disease status and survival.

There are several limitations in this study because it is retrospective. The number of patients was small enough that further analysis may yield additional possible adverse prognostic factors, such as ECS and PNI, which were not statistically significant in this study. Also difficult is to evaluate the importance of PORT in early oral tongue cancer because of the small number of irradiated patients. However, this study has summarized results of therapy targeting T_1-2_N_0-1 _OSCC in order to elucidate prognostic factors and improve postoperative clinical applications of RT.

## Conclusion

In T_1-2_N_0-1 _OSCC, factors that significantly associated with prognosis after primary surgery were poor tumor differentiation and deep invasion depths over 0.5 cm. PORT was not significantly related to clinical outcomes in T_1-2_N_0-1 _OSCC. Although not statistically significant, notably, no local failure occurred in the patients who received PORT in the high-risk group. PORT should therefore, be considered in early oral tongue cancer patients with high-risk pathologic features.

## Competing interests

The authors declare that they have no competing interests.

## Authors' contributions

SJS and KCK developed the ideas for these experiments, performed much of the work, and drafted the manuscript. JC, WSK, GEK, CGL, and ECC designed the study, collected the data and interpreted the data. SJS and JC performed the statistical analysis. All authors read and approved the final manuscript.

## References

[B1] Chen AY Myers JN Cancer of the oral cavity Dis Mon 2001 47 275 361 10.1067/mcd.2001.109374 11477373

[B2] Fein DA Mendenhall WM Parsons JT McCarty PJ Stringer SP Million RR Cassisi NJ Carcinoma of the oral tongue: a comparison of results and complications of treatment with radiotherapy and/or surgery Head Neck 1994 16 358 365 10.1002/hed.2880160410 8056581

[B3] Robertson AG Soutar DS Paul J Webster M Leonard AG Moore KP McManners J Yosef HM Canney P Errington RD Early closure of a randomized trial: surgery and postoperative radiotherapy versus radiotherapy in the management of intra-oral tumours Clin Oncol (R Coll Radiol) 1998 10 155 160 9704176 10.1016/s0936-6555(98)80055-1

[B4] Bernier J Domenge C Ozsahin M Matuszewska K Lefebvre JL Greiner RH Giralt J Maingon P Rolland F Bolla M Postoperative irradiation with or without concomitant chemotherapy for locally advanced head and neck cancer N Engl J Med 2004 350 1945 1952 10.1056/NEJMoa032641 15128894

[B5] Cooper JS Pajak TF Forastiere AA Jacobs J Campbell BH Saxman SB Kish JA Kim HE Cmelak AJ Rotman M Postoperative concurrent radiotherapy and chemotherapy for high-risk squamous-cell carcinoma of the head and neck N Engl J Med 2004 350 1937 1944 10.1056/NEJMoa032646 15128893

[B6] Lefebvre JL Coche-Dequeant B Buisset E Mirabel X Van JT Prevost B Management of early oral cavity cancer. Experience of Centre Oscar Lambret Eur J Cancer B Oral Oncol 1994 30B 216 220 10.1016/0964-1955(94)90095-7 7920170

[B7] Lapeyre M Bollet MA Racadot S Geoffrois L Kaminsky MC Hoffstetter S Dolivet G Toussaint B Luporsi E Peiffert D Postoperative brachytherapy alone and combined postoperative radiotherapy and brachytherapy boost for squamous cell carcinoma of the oral cavity, with positive or close margins Head Neck 2004 26 216 223 10.1002/hed.10377 14999796

[B8] Yuen AP Wei WI Wong YM Tang KC Elective neck dissection versus observation in the treatment of early oral tongue carcinoma Head Neck 1997 19 583 588 10.1002/(SICI)1097-0347(199710)19:7<583::AID-HED4>3.0.CO;2-3 9323146

[B9] Soutar DS McGregor IA The radial forearm flap in intraoral reconstruction: the experience of 60 consecutive cases Plast Reconstr Surg 1986 78 1 8 10.1097/00006534-198607000-00001 3725941

[B10] Rusthoven K Ballonoff A Raben D Chen C Poor prognosis in patients with stage I and II oral tongue squamous cell carcinoma Cancer 2008 112 345 351 10.1002/cncr.23183 18041071

[B11] Keum KC Chung EJ Koom WS Cho JH Cho SH Choi EC Lee CG Suh CO Kim GE Predictive value of p53 and PCNA expression for occult neck metastases in patients with clinically node-negative oral tongue cancer Otolaryngol Head Neck Surg 2006 135 858 864 10.1016/j.otohns.2006.02.011 17141074

[B12] Kligerman J Lima RA Soares JR Prado L Dias FL Freitas EQ Olivatto LO Supraomohyoid neck dissection in the treatment of T1/T2 squamous cell carcinoma of oral cavity Am J Surg 1994 168 391 394 10.1016/S0002-9610(05)80082-0 7977957

[B13] Fakih AR Rao RS Borges AM Patel AR Elective versus therapeutic neck dissection in early carcinoma of the oral tongue Am J Surg 1989 158 309 313 10.1016/0002-9610(89)90122-0 2802032

[B14] Spaulding CA Korb LJ Constable WC Cantrell RW Levine PA The influence of extent of neck treatment upon control of cervical lymphadenopathy in cancers of the oral tongue Int J Radiat Oncol Biol Phys 1991 21 577 581 1869456 10.1016/0360-3016(91)90673-r

[B15] Kirita T Okabe S Izumo T Sugimura M Risk factors for the postoperative local recurrence of tongue carcinoma J Oral Maxillofac Surg 1994 52 149 154 10.1016/0278-2391(94)90398-0 8295049

[B16] Al-Rajhi N Khafaga Y El-Husseiny J Saleem M Mourad W Al-Otieschan A Al-Amro A Early stage carcinoma of oral tongue: prognostic factors for local control and survival Oral Oncol 2000 36 508 514 10.1016/S1368-8375(00)00042-7 11036243

[B17] Arduino PG Carrozzo M Chiecchio A Broccoletti R Tirone F Borra E Bertolusso G Gandolfo S Clinical and histopathologic independent prognostic factors in oral squamous cell carcinoma: a retrospective study of 334 cases J Oral Maxillofac Surg 2008 66 1570 1579 10.1016/j.joms.2007.12.024 18634942

[B18] Lee N Xia P Fischbein NJ Akazawa P Akazawa C Quivey JM Intensity-modulated radiation therapy for head-and-neck cancer: the UCSF experience focusing on target volume delineation Int J Radiat Oncol Biol Phys 2003 57 49 60 10.1016/S0360-3016(03)00583-2 12909215

[B19] Hoppe BS Wolden SL Zelefsky MJ Mechalakos JG Shah JP Kraus DH Lee N Postoperative intensity-modulated radiation therapy for cancers of the paranasal sinuses, nasal cavity, and lacrimal glands: technique, early outcomes, and toxicity Head Neck 2008 30 925 932 10.1002/hed.20800 18302261

[B20] Gomez DR Zhung JE Gomez J Chan K Wu AJ Wolden SL Pfister DG Shaha A Shah JP Kraus DH Intensity-modulated radiotherapy in postoperative treatment of oral cavity cancers Int J Radiat Oncol Biol Phys 2009 73 1096 1103 18707827 10.1016/j.ijrobp.2008.05.024

